# Cumulative prevalence of biological and social risk factors at birth in a city in São Paulo

**DOI:** 10.1590/1980-220X-REEUSP-2021-0328

**Published:** 2022-03-23

**Authors:** Aline Adryane Morishigue Bássiga da Cruz, Lucas Cardoso dos Santos, Michelle Cristine de Oliveira Minharro, Rúbia Aguiar Alencar

**Affiliations:** 1 Universidade Estadual Paulista, Faculdade de Medicina de Botucatu, Departamento de Enfermagem, Botucatu, SP, Brazil.; 2 Universidade Nove de Julho, Faculdade Marechal Rondon, Botucatu, SP, Brazil.

**Keywords:** Primary Health Care, Infant, Postmature, Risk Factors, Atención Primaria de Salud, Posmaduro, Factores de Riesgo, Atenção Primária à Saúde, Criança Pós-Termo, Fatores de Risco

## Abstract

**Objective:**

to identify the cumulative prevalence of biological and social risk factors at birth.

**Method:**

a cross-sectional study, with retrospective data collection, carried out with live births in a medium-sized city, from January 2018 to July 2020. A database was used with information aimed at identifying social and biological risks after birth, assessed descriptively.

**Results:**

the sample consisted of 4,480 newborns, of which 78.9% were classified as at usual risk, and 21.1% as at risk. The cumulative prevalence showed that most newborns had more than one risk factor, with biological risks being the most prominent: need for admission to Intensive Care Unit, birth with less than 37 weeks of gestation and weight less than 2,500 g. Among the social risks, the following stand out: newborns who had a dead sibling aged less than 5 years old; head of family without income; mothers under 16 years old and who did not undergo prenatal care. The biological risk rate was 7.39 times higher than the social risk rate.

**Conclusion:**

the cumulative prevalence of the risks found was significant, with a considerable part of the sample presenting some biological risk.

## RESUMO

**Objetivo:**

identificar a prevalência cumulativa de fatores de riscos biológicos e sociais ao nascer.

**Método:**

estudo transversal, com coleta retrospectiva de dados, realizado com os nascidos vivos de um município de médio porte, no período de janeiro de 2018 a julho de 2020. Utilizou-se banco de dados com informações voltadas para a identificação de riscos sociais e biológicos após o nascimento, avaliados de forma descritiva.

**Resultados:**

a amostra foi composta por 4.480 recém-nascidos, sendo 78,9% classificados como bebês de risco habitual, e 21,1%, como de risco. A prevalência cumulativa evidenciou que a maior parte dos recém-nascidos possuía mais de um fator de risco, sendo os riscos biológicos com maior destaque: a necessidade de internação em Unidade de Terapia Intensiva, nascimento com menos de 37 semanas de gestação e peso menor que 2.500 g. Dentre os riscos sociais, evidencia-se: recém-nascidos que tiveram irmão morto com idade menor que 5 anos de idade; chefe de família sem renda; mães com menos de 16 anos e que não realizaram o pré-natal. A taxa de risco biológico foi 7,39 vezes maior que a taxa de risco social.

**Conclusão:**

a prevalência cumulativa dos riscos encontrados foi significativa com considerável parte da amostra, apresentando algum risco biológico.

## RESUMEN

**Objetivo:**

identificar la prevalencia acumulada de factores de riesgo biológicos y sociales al nacer.

**Método:**

estudio transversal, con recolección de datos retrospectiva, realizado con nacidos vivos en un municipio de mediano porte, de enero de 2018 a julio de 2020. Se utilizó una base de datos con información destinada a identificar riesgos sociales y biológicos después del nacimiento, evaluados de forma descriptiva.

**Resultados:**

la muestra estuvo constituida por 4.480 recién nacidos, de los cuales el 78,9% fueron clasificados como de riesgo habitual y el 21,1% como de riesgo. La prevalencia acumulada mostró que la mayoría de los recién nacidos tenían más de un factor de riesgo, siendo los biológicos los más destacados: necesidad de hospitalización en Unidad de Cuidados Intensivos, nacimiento con menos de 37 semanas de gestación y peso inferior a 2.500 g. Entre los riesgos sociales se destacan: los recién nacidos que tuvieron un hermano menor de 5 años muerto; cabeza de familia sin ingresos; madres menores de 16 años y que no realizaron control prenatal. La tasa de riesgo biológico fue 7,39 veces superior a la tasa de riesgo social.

**Conclusión:**

la prevalencia acumulada de los riesgos encontrados fue significativa, presentando una parte considerable de la muestra algún riesgo biológico.

## INTRODUCTION

The neonatal period is considered the most vulnerable for children under the age of five, in which one third of all neonatal deaths occur on the first day and three-quarters do not survive the first week of life, accounting for between 45–49% of all deaths before the age of five^(1)^.

In this context, neonatal and infant mortality represent a global concern. Worldwide, it is estimated that 3.9 million deaths occurred in 2019 alone, of which 2.4 million occurred within the first month of life, with an estimated neonatal mortality rate of 17 deaths per 1,000 live births^(1)^.

In Brazil, infant mortality rate has been falling in recent decades, where infant mortality increased from 29 deaths per thousand live births in 2000 to an estimated 12 deaths in 2019^(1,2)^.

However, there is still an alarming number of deaths from preventable causes, especially in the neonatal period, which is commonly associated with inadequate care for pregnant women and newborns (NB). It is estimated that between 2020 and 2030 there may be 48 million deaths in children under five years of age, in which half of these deaths will be NB, which can be prevented through high coverage and quality of prenatal care, specialized care during childbirth and care. in the postnatal period for the mother-baby binomial, with differentiated attention to premature NB^(1)^.

In this context, initiatives were implemented in Brazil to strengthen the recommended lines of care and assist in the comprehensive mother-baby binomial care. Among these initiatives are the *Rede Cegonha*, the Brazilian National Policy for Comprehensive Care for Children’s Health (*Política Nacional de Atenção Integral à Saúde da Criança*), the Humanization of Prenatal and Birth Program (*Programa de Humanização do Pré-Natal e Nascimento*), the Brazilian National Pact for the Reduction of Maternal and Neonatal Mortality (*Pacto Nacional pela Redução da Mortalidade Materna e Neonatal*) and the Agenda of Commitments for the Comprehensive Health of the Child and Child Mortality Reduction (*Agenda de Compromissos para a Saúde Integral da Criança e Redução da Mortalidade Infantil*)^(3)^.

Primary Health Care (PHC), especially Family Health Strategy (FHS), is highlighted, as it plays a fundamental role in the care of pregnant women, mothers, NB and their families^(4,5)^. Brazilian and Canadian researchers point to a significant reduction in infant mortality, ranging from 3–9% in the second year, after the implementation of FHS in the cities, regardless of socioeconomic status. This impact is even greater in the long term, reaching values between 6.7–14% in the third year^(6)^.

At-risk newborns (RNB) have factors that predispose to unfavorable conditions for development, in addition to having a higher rate of morbidity and mortality and developing disabling sequelae during life^(7,8)^.

The Ministry of Health (MoH) suggests criteria for the identification of RNB: low birth weight (<2,500 g); delivery at less than 37 weeks of gestational age (GA), 5-minute Apgar <7; hospital admissions; teenage mother (<20 years); mother with low education (<8 years of schooling); residence in risk area; history of death of children under 5 years of age in the family; low socioeconomic level; and explicitly unwanted child^(8)^.

However, regardless of the criteria used to classify the NB as at risk or not, this population must be monitored in a differentiated, systematic and frequent way, as they are more likely to have jaundice, respiratory failure, tachypnea, apnea, neonatal infections, intracranial hemorrhage, heart disease, anemia, gastroesophageal reflux, seizures, hypoglycemia, and other conditions that compromise healthy development^(9)^.

NB surveillance and health care assessment is essential to reduce neonatal and infant mortality, since care is influenced by multiple factors, such as investments by managers and accountability by health professionals, especially in intervention timely, when risk situations are identified. According to a study that carried out a systematic review with meta-analysis on the main risk factors for neonatal mortality in Brazil, it was identified that these risks are modifiable, depending on the timely intervention^(10)^.

Therefore, considering the reality of each city and each area covered by the health units, it is necessary to know the biological and social risks that involve the NB. It is believed that, in this way, it is possible to plan care and develop actions based on scientific evidence, to ensure a positive outcome for NB.

Therefore, it is necessary to improve the quality of care provided by the services to NB and consider primary prevention as one of the main objectives in these actions, allowing better monitoring and monitoring of risks^(11)^.

The research is justified by the fact that, although the literature discusses NB’s social and biological risks^(7,9,10)^, there are still challenges to be overcome in order to have comprehensive NB care, since there are gaps between programmatic guidelines and the capillarity of public health policies aimed at this population, including as a challenge the real knowledge of individuals’ and families’ needs for planning health actions^(3)^.

Based on the above, the study aims to identify the cumulative prevalence of biological and social risk factors at birth in a city in São Paulo.

## METHOD

### Type of Study

This is a cross-sectional study with retrospective data collection.

### Local

It was carried out in a medium-sized city in the countryside of the state of São Paulo, with an estimated population of 148,130 inhabitants, and an infant mortality rate of 13.99 deaths per thousand live births in 2017, totaling 24 deaths from children under one year^(2)^.

The city has two linked maternity hospitals, respectively, a teaching hospital of medium and high complexity, a reference for 68 cities in the region, and a private institution. In the context of PHC, it has 22 services distributed in Basic Health Units in the traditional model, Family Health and School Health Centers.

### Population

All live births in the city were included in the study, from January 2018 to July 2020.

### Data Collection

The data used in this research are secondary to a database of the city that contains information from the RNB Surveillance Form, filled out for all NB after birth.

This form uses the criteria suggested by the MoH, with adaptations to the city’s reality, such as maternal age, in which maternal age under 16 is considered a risk, taking into account factors that increase the risks of teenage pregnancy according to the Brazilian Society of Pediatrics, since the literature does not provide an age consensus to define teenage pregnancy, ranging from 15 to 20 years^(8,12,13)^.

Filling out the form was carried out through an interview with the mother during the hospitalization period (postpartum), or through secondary data from medical records, when the mother is not present during the visit of employees of the Municipal Health Department to maternity hospitals in the city.

The study variables were: notifiable diseases of the mother or NB; biological risk (birth weight < 2,500 g, GA at birth less than 37 weeks, major or multiple congenital malformation/genetic disease, admission to the Intensive Care Unit (ICU)/Intermediate Care Unit (ICU), 5-minute Apgar less than 7); and social risk (dead sibling under five years old, maternal age below 16 years old, mother unable to take care of child due to psychiatric problems, chemical dependency, imprisonment, illness or other problem, illiterate mother, mother without a partner and without family support, mother without prenatal segment, head of family without income).

For the NB to be considered at risk, the city considers that it has at least one biological risk and/or two or more social risks.

### Data Analysis and Treatment

Data were tabulated in Microsoft Excel^®^ spreadsheets. For the statistical analysis processing, the data were transported to the Stata program (statistics/data analysis). Descriptive analysis was performed using absolute and relative numbers with simple frequency, mean and standard deviation. The variable called risk rate was also calculated, which expresses the mean number of risk conditions per NB in each of the PHC services, which can range from 0 to infinity, and the higher its value, the greater the mean number of risk conditions per NB.

### Ethical Aspects

The research was carried out in accordance with Resolution 466 of December 12, 2012, and approved by the Institutional Review Board of the *Faculdade de Medicina de Botucatu*, under Opinion 4,063,497, on June 2, 2020. The application of the Informed Consent Form was waived, as there was no contact with people of any kind, thus preserving their identities.

## RESULTS

The sample consisted of all live births during the study period, totaling 4,480 NB, of which 3,533 (78.9%) were classified as habitually risky infants, 947 (21.1%) as RNB. 0.89% (n = 40) had some disease of compulsory notification, and of these, 0.3% (n = 13) did not have the notification disease specified in the form (Table 1).

**Table 1.** Distribution of variables, biological and social risks of a total of 4,480 newborns, from 2018 to 2020. Botucatu, SP, Brazil, 2020.

		n	%
**Variables**	**Risk stratification**		
	Usual risk	3533	78.9
	At-risk newborn	947	21.1
	**Compulsory notification disease**		
	No	4440	99.1
	Without the description of the reported disease	13	0.3
	Syphilis	19	0.4
	HIV	1	0.0
	HIV under investigation	1	0.0
	Toxoplasmosis	4	0.1
	Syphilis and Hepatitis C	1	0.0
	Gonorrhea and HPV	1	0.0
**Biological risks**	**Death at birth**		
	No	4478	99.9
	Yes	2	0.044
	**Weight < 2,500 g**		
	No	4089	91.3
	Yes	391	8.7
	**Gestational age < 37 weeks**		
	No	4035	90.1
	Yes	445	9.9
	**Major or multiple congenital malformation/genetic disease**		
	No	4427	98.8
	Yes	53	1.2
	**Intensive Care Unit Admission**		
	No	3989	89.0
	Yes	491	11.0
	**5-minute Apgar less than 7**		
	No	4431	98.9
	Yes	49	1.1
**Social risks**	**Brother killed < 5 years**		
	No	4411	98.5
	Yes	69	1.5
	**Maternal age under 16 years**		
	No	4449	99.3
	Yes	31	0.7
	**Mother unable to care for child**		
	No	4470	99.8
	Yes	10	0.2
	**Illiterate mother**		
	No	4477	99.9
	Yes	3	0.1
	**Mother without a partner and without family support**		
	No	4464	99.6
	Yes	16	0.4
	**No prenatal care follow-up**		
	No	4453	99.4
	Yes	27	0.6
	**Head of family without income**		
	No	4417	98.6
	Yes	63	1.4

In relation to biological risks, it is noteworthy that 11% (n = 491) of NB required ICU admission; 9.9% (n = 445) were born less than 37 weeks old; and 8.7% (n = 391) had a weight lower than 2,500 g. Among the social risks, it was observed that 1.5% (n = 69) of NB had a dead brother under the age of five; 1.4% (n = 63) had the head of family without income; 0.7% (n = 31) were mothers under 16 years of age; and 0.6% (n = 27) did not undergo prenatal care follow-up (Table 1).

In Table 2, it is possible to assess the total number of NB distributed in Primary Care Health services, in addition to identifying how many NB were classified as RNB and their cumulative risks, and the sum of risks in most services was greater than the number of RNB. Moreover, it is evident that in the city the mean and standard deviation of biological risk (0.32 ± 0.74) were higher than those of social risk (0.05 ± 0.26).

**Table 2.** Distribution of the mean and standard deviation of biological and social risks by health unit and city, from 2018 to 2020. Botucatu, SP, Brazil, 2020.

Health units	Total n° of NB	N° of NB–usual risk	N° of RNB	Cumulative risk*	Number of risks	
					Biological risk mean ± SD (min–max)	Social risk mean ± SD (min–max)
**1**	338	277	61	100	0.26 ± 0.64 (0–4)	0.03 ± 0.19 (0–2)
**2**	417	337	80	130	0.27 ± 0.69 (0–4)	0.04 ± 0.19 (0–1)
**3**	1	0	1	1	1 ± (1–1)	0 ± (0–0)
**4**	406	317	89	145	0.29 ± 072 (0–4)	0.06 ± 0.24 (0–1)
**5**	240	188	52	98	0.35 ± 0.80 (0–4)	0.05 ± 0.29 (0–3)
**6**	197	162	35	68	0.28 ± 0.66 (0–3)	0.07 ± 0.52 (0–7)
**7**	207	162	45	91	0.39 ± 0.86 (0–5)	0.05 ± 0.21 (0–1)
**8**	97	71	26	40	0.46 ± 1.00 (0–4)	0.05 ± 0.22 (0–1)
**9**	16	15	1	1	0.06 ± 025 (0–1)	0.00 ± 0.00 (0–0)
**10**	215	175	40	78	0.34 ± 0.81 (0–4)	0.02 ± 0.15 (0–1)
**11**	144	109	35	64	0.34 ± 0.76 (0–4)	0.10 ± 0.62 (0–7)
**12**	238	186	52	84	0.32 ± 0.69 (0–3)	0.04 ± 0.22 (0–2)
**13**	378	284	94	144	0.32 ± 071 (0–4)	0.06 ± 027 (0–3)
**14**	101	79	22	43	0.37 ± 0.79 (0–3)	0.06 ± 0.23 (0–1)
**15**	217	164	53	95	0.41 ± 0.82 (0–3)	0.03 ± 016 (0–1)
**16**	285	230	55	91	0.27 ± 068 (0–4)	0.05 ± 0.23 (0–2)
**17**	256	199	57	93	0.32 ± 070 (0–5)	0.05 ± 0.21 (0–1)
**18**	156	124	32	62	0.36 ± 081 (0–4)	0.04 ± 019 (0–1)
**19**	150	143	27	49	0.28 ± 0.68 (0–3)	0.05 ± 0.24 (0–2)
**20**	309	250	59	111	0.31 ± 073 (0–4)	0.05 ± 0.22 (0–2)
**21**	112	81	31	49	0.38 ± 0.77 (0–3)	0.05 ± 0.22 (0–1)
**City**	**4.480**	**3533**	**947**	**1637**	**0.32 ± 0.74 (0–5)**	**0.05 ± 0.26 (0–7)**

^*^ Whereas the NB may have more than one risk factor.

In the city, each NB presented, on average, 0,397 risk conditions in general, being 0.351 biological and 0.045 social per NB, which demonstrates a higher occurrence of biological risks.

The biological risk rate was 7.39 times higher than the social risk rate in the city, according to Table 3.

**Table 3.** Distribution of biological, social and general risk rates of each health unit, from 2018 to 2020. Botucatu, SP, Brazil, 2020.

Health units	Biological risk rate	Social risk rate	General risk rate	Rate ratio
**1**	0.263	0.033	0.296	7.97
**2**	0.273	0.038	0.312	7.18
**3**	1.000	0.000	1.000	–
**4**	0.293	0.064	0.357	4.58
**5**	0.354	0.054	0.408	6.56
**6**	0.279	0.066	0.345	4.23
**7**	0.391	0.048	0.440	8.15
**8**	0.464	0.052	0.515	8.92
**9**	0.063	0.000	0.063	–
**10**	0.340	0.023	0.363	14.78
**11**	0.340	0.104	0.444	3.27
**12**	0.315	0.042	0.357	7.50
**13**	0.320	0.061	0.381	5.25
**14**	0.366	0.059	0.426	6.20
**15**	0.410	0.028	0.438	14.64
**16**	0.267	0.053	0.319	5.04
**17**	0.316	0.047	0.363	6.72
**18**	0.359	0.038	0.397	9.45
**19**	0.280	0.047	0.327	5.96
**20**	0.314	0.045	0.359	6.98
**21**	0.384	0.054	0.438	7.11
**City**	**0.351**	**0.045**	**0.397**	**7.39**

## DISCUSSION

The present study allowed the identification of the biological and social risks of NB, as well as the cumulative risk of babies at birth. Although most NB did not present any risk (78.9%), 31.9% and 4.9% presented, respectively, some biological or social risk.

Biological risks were distributed so that the need for ICU admission, birth less than 37 weeks of gestation and weight less than 2,500 g were highlighted, compared to congenital, multiple or genetic malformation and fifth-minute Apgar less than seven.

These findings converge with results from the national and international literature, in which analysis conducted in Turkey found that GA less than 37 weeks of gestation and birth weight less than 2,500 g were among the most important variables related to long periods of hospitalization of NB^(14)^. In a Brazilian study, prematurity, low birth weight and the need for ICU admission were highlighted among biological risks^(15)^.

Among the diseases of compulsory notification identified, there is a predominance of the notification of congenital syphilis, with 4.5 babies reported for every thousand live births, being above the goal established by the Pan American Health Organization (PAHO), which recommends an incidence rate of 0.5 cases for every 1,000 live births^(16)^.

The high rate of congenital syphilis is a national reality, as shown by a study that mapped clusters of the disease in the country, which contradicts the downward trend found on the world stage^(17)^.

This scenario probably reflects the socioeconomic fragility of most developing countries on the American continent, translated into lack of resources and inequality in their distribution, representing two important social determinants in the health-disease process and demonstrating a relationship between biological and social risks^(17)^.

In the context of this study, the most prevalent social risks were represented by NB who had a dead sibling under the age of five, had the head of family without income, had mothers under 16 years of age and mothers who did not undergo prenatal care.

In a follow-up program for RNB, it was found that most families had a mean income of 2.4 minimum wages, that the families had an employed head of family and that the NB’s mother did not have a job at the time^(14)^. In a study carried out in the United States, it was concluded that low socioeconomic status has a direct impact on increased risk for prematurity^(18)^.

The findings of this research related to the cumulative prevalence showed that most NB had more than one risk factor. Although NB needed to present at least two social risks to be classified as RNB, most of the total RNB assessed presented more than one risk, regardless of being social or biological, which resulted in a significant cumulative prevalence.

This finding agrees with a European study, which showed the association of two or more socioeconomic risks related to a considerable increase in the occurrence of prematurity and low birth weight, demonstrating a cumulative effect of risks and linking social and biological factors^(19)^.

In this scenario, PHC plays a fundamental role in monitoring NB after birth, as it plays the role of coordinator of the Health Care Network (RAS – *Rede de Atenção à Saúde*), reducing possible barriers to access and ensuring comprehensiveness and continuity of care between the different levels of care, which can be considered the main strategy for rearranging the care model^(20)^.

Carrying out health surveillance actions developed by PHC, especially by FHS, can reduce neonatal and infant mortality through guidance to postpartum women, carrying out neonatal tests, strengthening the bond between the NB’s family and the health service, home visits, vaccination and assessment of growth and development, in addition to carrying out a situational diagnosis of the territory^(21)^. In this context, health surveillance becomes essential for care planning.

A systematic review, which synthesized the main evidence on FHS and its impact in Brazil, concluded that the implementation of FHS is significant for improving the population’s health, contributing to the reduction of infant mortality. In cities with coverage greater than 70% in four years of FHS, there may be a reduction in the neonatal mortality rate between 11% and 44%, and post-neonatal mortality, between 17% and 31%^(22)^.

It is noteworthy that the increase in FHS coverage by 10% corresponds to a decrease in infant mortality from 0.4% to 4.6% depending on the period and units assessed^(22)^. These results corroborate with others found in the literature. Canadian research and Brazilian research concluded that the reduction in mortality varies between 0.8% and 66%, depending on the coverage of FHS in the cities^(23)^.

Based on this assumption, it was possible, through the present study, to show the distribution of biological and social risks by health unit, even though this was not the initial objective, since, to arrive at the total values, it was necessary to individualize the data found in each service. It is emphasized that the risk rate evidenced per health unit expresses the mean number of risk conditions per NB, which allows each unit to develop specific actions related to NB health surveillance in its area of coverage.

Professionals and managers should use the territory’s health indicators as a tool for planning health and social actions and needs corresponding to the type of risk identified, together with the assisted population^(24)^.

Considering the results obtained and as implications for practice, it is expected that this research can foster debate with the city’s political authorities, through discussion between health professionals from the various RAS services, seeking the integration and exchange of experiences between health professionals and municipal managers to strengthen actions, with the intention of improving NB health surveillance. It is believed that the present study may also provide opportunities for other cities to identify the cumulative prevalence of biological and social risk factors at birth as well as the risk rate.

Among the limitations of this study, there is the use of secondary data, which made it possible to assess only the information extracted from them, not being possible to classify maternal age over 35 years as a risk, and the inclusion of variables such as mean family income and type of delivery. Furthermore, the authors emphasize that the study reflects the local reality; thus, generalization and comparison with other cities should consider the characteristics of a medium-sized city.

## CONCLUSION

It was possible to identify that approximately 11% of the sample presented some biological risk and that the cumulative prevalence of the risks found in this study is significant. Prematurity, low birth weight and ICU admission were the biological risks that most influenced NB. Regarding the social risks found, NB who had a dead sibling under the age of five stand out, had the head of family without income, had mothers under 16 years of age and mothers who did not undergo prenatal care. However, the biological risk rate was 7.39 times higher than the social risk rate.

To advance in NB surveillance and health care, with the objective of reducing neonatal and infant mortality, it is necessary to know the biological and social risks present in the reality of each city. This knowledge enables the planning and elaboration of actions based on scientific evidence.

Future studies that contemplate the reality of other cities are recommended, aiming to deepen the knowledge of the epidemiological situation in the country and to structure management tools and planning of health actions that help in NB care.

## ASSOCIATE EDITOR

Maria Luiza Gonzalez Riesco
